# IDH1 mutation is associated with lower expression of VEGF but not microvessel formation in glioblastoma multiforme

**DOI:** 10.18632/oncotarget.24536

**Published:** 2018-02-20

**Authors:** Jiří Polívka, Martin Pešta, Pavel Pitule, Ondřej Hes, Luboš Holubec, Jiří Polívka, Tereza Kubíková, Zbyněk Tonar

**Affiliations:** ^1^ Department of Histology and Embryology, Charles University, Faculty of Medicine in Pilsen, 306 05 Pilsen, Czech Republic; ^2^ Biomedical Centre, Charles University, Faculty of Medicine in Pilsen, 306 05 Pilsen, Czech Republic; ^3^ Department of Neurology, Faculty Hospital Pilsen, 304 60 Pilsen, Czech Republic; ^4^ Department of Biology, Charles University, Faculty of Medicine in Pilsen, 306 05 Pilsen, Czech Republic; ^5^ Department of Pathology, Faculty Hospital Pilsen, 304 60 Pilsen, Czech Republic

**Keywords:** glioblastoma multiforme, isocitrate dehydrogenase, microvessel, biomarkers, microvascularity

## Abstract

**Introduction:**

Glioblastoma multiforme (GBM) represents the most malignant primary brain tumor characterized by pathological vascularization. Mutations in isocitrate dehydrogenases 1 and 2 (IDH1 and IDH2) were observed in GBM. We aimed to assess the intra-tumor hypoxia, angiogenesis and microvessel formation in GBM and to find their associations with IDH1 mutation status and patients prognosis.

**Methods:**

52 patients with a diagnosis of GBM were included into the study. IDH1 R132H mutation was assessed by RT-PCR from FFPE tumor samples obtained during surgery. The expression of markers of hypoxia (HIF2α), angiogenesis (VEGF), tumor microvascularity (CD31, CD34, vWF, CD105), and proliferation (Ki-67) were assessed immunohistochemically (IHC). IDH1 mutation and IHC markers were correlated with the patient survival.

**Results:**

20 from 52 GBM tumor samples comprised IDH1 R132H mutation (38.5%). The majority of mutated tumors were classified as secondary glioblastomas (89.9%). Patients with IDH1 mutated tumors experienced better progression-free survival (P = 0.037) as well as overall survival (P = 0.035) compared with wild type tumors. The significantly lower expression of VEGF was observed in GBM with IDH1 mutation than in wild type tumors (P = 0.01). No such association was found for microvascular markers. The increased expression of newly-formed microvessels (ratio CD105/CD31) in tumor samples was associated with worse patient’s progression-free survival (P = 0.026).

**Summary:**

No increase in HIF/VEGF-mediated angiogenesis was observed in IDH1-mutated GBM compared with IDH1 wild type tumors. The histological assessment of the portion of newly-formed microvessels in tumor tissue can be used for the prediction of GBM patient’s prognosis.

## INTRODUCTION

Glioblastoma multiforme (GBM) represents the most common and most malignant primary brain tumor in adults with an incidence of 3-4/100,000/year [1]. The prognosis of patients diagnosed with GBM remains extraordinary poor with the median survival only 12.1–14.6 months [2, 3]. Moreover, no more than 3–5% of patients survive longer than 3 years [1, 4]. Genomics and epigenomics of GBM, together with other glial tumors, were extensively researched during the last decade that led into the identification of several abnormalities in major cellular signaling pathways with a diversity of mutated genes in gliomas [5–7]. The essential role among them represents the metabolic enzyme isocitrate dehydrogenase (IDH) [5, 8]. Isocitrate dehydrogenases (three isoforms IDH1, IDH2, and IDH3) catalyze the oxidative carboxylation of isocitrate to alpha-ketoglutarate and reduce nicotinamide adenine dinucleotide phosphate (NADP) to NADPH, which is necessary for the regeneration of reduced glutathione that serves as the main cellular antioxidant [9]. The mutated IDH acquires aberrant and oncogenic function, namely the conversion of alpha-ketoglutarate to the oncometabolite 2-hydroxyglutarate (2-HG), which subsequently leads to genome-wide epigenetic changes in human gliomas and their progression [10, 11]. The mutations in IDH1/2 represent also an independent and important GBM prognostic factor [12–16] and their routine assessment should be the standard in the clinical management of patients with gliomas (including GBM) according to the recently updated World Health Organization (WHO) 2016 classification of CNS tumors and European Association for Neuro-Oncology (EANO) guidelines on the diagnosis and treatment of glial tumors [17, 18].

The GBM microenvironment and its involvement in cancer development and progression was extensively studied, especially tumor angiogenesis and neovascularization [19, 20]. GBM is highly vascularized tumor with substantial microvascular proliferation surrounding necrotic areas [17, 21, 22]. There is still a relative lack of studies, above that with inconclusive results, which assess the relation of GBM genetics and especially IDH mutation status with tumor angiogenesis and microvessel formation. Originally, it was hypothesized that the aberrantly produced 2-HG by mutated IDH may compete with alpha-ketoglutarate and inhibit the prolyl hydroxylase (PHD), which in turn can lead to the pathological stabilization of hypoxia inducible factor (HIF) and induction of the expression of the major proangiogenic vascular endothelial growth factor (VEGF) and thus initiation of tumor angiogenesis [23, 24]. However, subsequent studies did not prove this hypothesis [25, 26]. Above that, more recent studies in human astrocytes, colorectal and erythroleukemia cell lines found the negative association between IDH mutations and HIF and VEGF expression [27, 28]. The conclusive information about the association of IDH mutations and tumor microvascular proliferation would be of a great clinical importance in relation to the development of targeted anticancer and antiangiogenic therapy for malignant gliomas [29]. Moreover, hypoxia may play the key role in the development and progression of the malignancies in CNS as has been recently presented in the context of the aggressive metastatic disease triggered by certain subtypes of breast cancer [30–33].

The aim of this study was to examine the hypoxia-related angiogenesis and tumor-specific microcirculation assessed by immunohistochemistry in the tissue samples obtained during surgery from patients with newly-diagnosed GBM, and to find the relationship between tumor microcirculation and IDH1 mutation status in the same patients. GBM microcirculation was assessed by histological quantification of expression of pan-endothelial markers (CD31, CD34, and von Willebrand factor – vWF) as well as the marker of newly-formed microvessels (CD105 - endoglin) together with the expression of the major proangiogenic vascular endothelial growth factor (VEGF) and the brain-specific activator of angiogenesis in the hypoxic condition – hypoxia inducible factor 2 alpha (HIF2α). The proliferative activity (Ki-67 antigen expression) was also assessed and correlated with GBM microcirculation. The prognostic significance of these markers and IDH1 mutation status in relation to patient’s progression-free survival (PFS) and overall survival (OS) was examined as well.

## RESULTS

### The occurrence of IDH1 R132H mutation and its relation to patients’ survival

The IDH1 R132H mutation was detected in the tissue samples from 20 GBM patients (38.5%), whereas the IDH1 wild-type tumor was observed in remaining 32 patients (61.5%). Because of the relatively high portion of IDH1 mutated tumors, the separation of primary and secondary GBM (progressing from the low-grade gliomas) was performed on the basis of clinical information from the patient history, where possible. The diagnosis of secondary GBM was established if there was a history of previously assessed low grade glioma, or if the patient suffered from presumably tumor corresponding neurological symptomatology (epileptic seizures, focal neurological deficit) at least 6 months before the final GBM diagnosis. Based on these criteria, IDH1 R132H mutation was detected in 4 from 34 primary GBMs (11.8%), whereas the majority of secondary GBMs comprised the mutation (16 of 18, 88.9%) (Table 1).

**Table 1:** The distribution of IDH1 R132H mutated tumors among primary and secondary glioblastomas

Mutation status	Primary glioblastoma (n=34)	Secondary glioblastoma (n=18)
IDH1 R132H	4 (11.8 %)	16 (88.9 %)
IDH1 wild type	30 (88.2 %)	2 (11.1 %)

**Figure 1:**
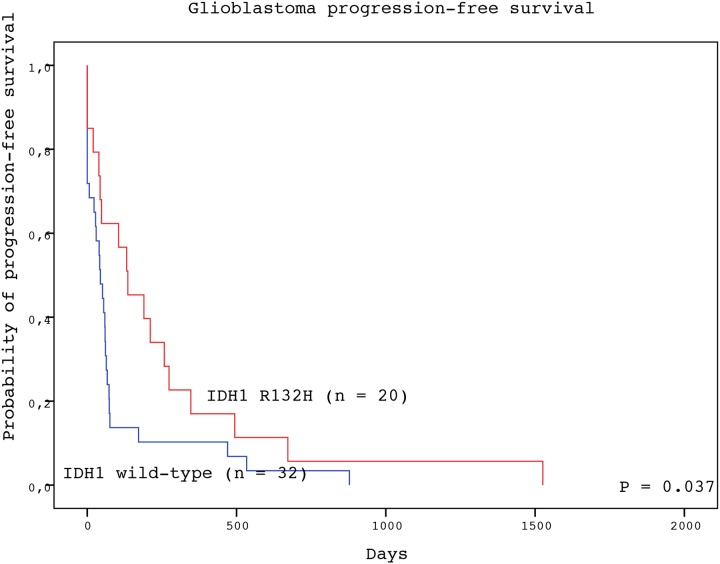
Progression-free survival of GBM patients with IDH1 R132H-mutated (red line) and IDH1 wild type (blue line) tumors (log-rank test)

**Figure 2:**
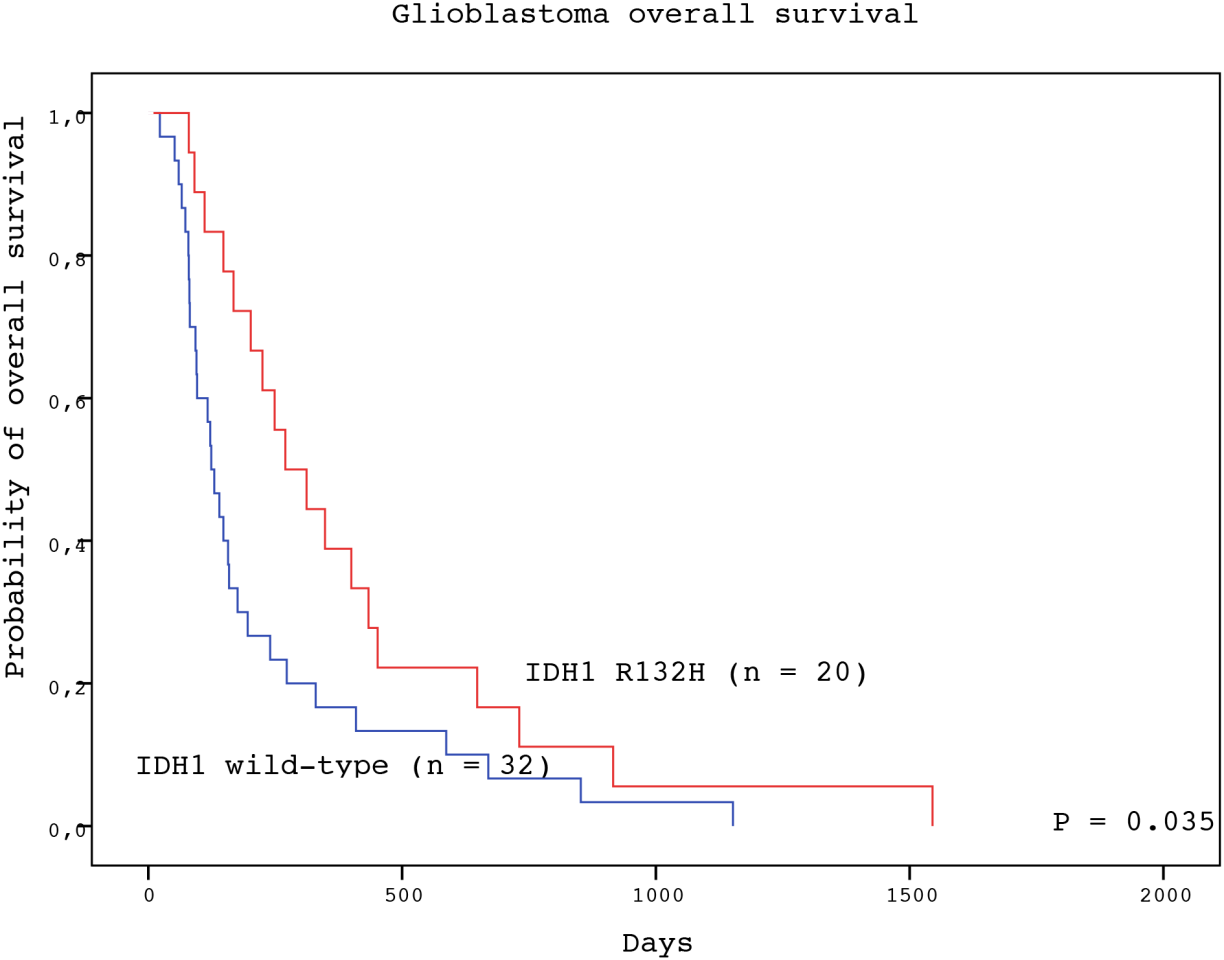
Overall survival of GBM patients with IDH1 R132H-mutated (red line) and IDH1 wild type (blue line) tumors (log-rank test)

The detection of IDH1 R123H mutation in the tumor tissue represented a significant positive prognostic factor for both PFS and OS of GBM patients (Table 2). Subjects with GBM positive for IDH1 R132H mutation experienced longer median PFS than patients with wild-type tumors (136 vs. 44 days; P = 0.037, log-rank test) (Figure 1). Likewise, patients with IDH1 R132H positive tumors had significantly longer median OS than those without the mutation (270 vs. 124 days; P = 0.035, log-rank test) (Figure 2). In the multivariate analysis, the IDH1 R132H mutation remained positive prognostic factor for OS (HR = 0.433, P = 0.011) but not PFS (HR = 0.560, P = 0.092) of GBM patients. The other significant prognostic factors in multivariate analysis were age at diagnosis (P = 0.002) and treatment after surgery (P = 0.003) (Table 3).

**Table 2:** Progression-free survival and overall survival of GBM patients in relation to the IDH1 mutation status

Survival analysis	Number of patients	Median [days] (95% Cl)	P value (log-rank)
**Progression-free Survival**			
IDH1 R132H	20	136 (22-249)	
IDH1 wild type	32	44 (17-71)	0.037
**Overall Survival**			
IDH1 R132H	20	270 (139-400)	
IDH1 wild type	32	124 (93-155)	0.035

**Table 3:** Multivariate Cox proportional hazards regression for progression free survival (PFS) and overall survival (OS) of patients with GBM

Characteristics	PFS HR (95% CI)	P value	OS HR (95% CI)	P value
**Age**				
< 60	1.000	0.088	1.000	**0.002**
≥ 60	1.945 (0.907 – 4.172)		3.691 (1.606 – 8.484)	
**Gender**				
Female	1.000	0.936	1.000	0.162
Male	0.974 (0.513 – 1.851)		0.632 (0.332 – 1.203)	
**KPS**				
< 90	1.000	0.607	1.000	0.319
≥ 90	0.85 (0.458 – 1.578)		0.726 (0.387 – 1.363)	
**Treatment**				
Surgery alone	1.000	**<0.001**	1.000	**0.003**
Surgery + RT/CT	0.241 (0.112 – 0.519)		0.343 (0.168 – 0.699)	
**IDH1 mutation status**				
IDH1 wild type	1.000	0.092	1.000	**0.011**
IDH1 R132H	0.560 (0.285 – 1.1)		0.433 (0.227 – 0.823)	

*PFS* – progression free survival; *OS* – overall survival; *HR* – hazard ratio; *CI* - confidence interval; *KPS* - Karnofsky performance score; *RT* - radiotherapy; *CT* – chemotherapy.

### Mutual correlations among microvascular, angiogenesis, hypoxia, and proliferation markers and other clinicopathological characteristics

The histologically quantified expression of microvascular endothelial markers (CD31, CD34, vWF) together with markers of active endothelia (CD105), proportions of newly-formed microvessels (ratios CD105/CD31, CD105/CD34 and CD105/vWF), intratumor hypoxia (HIF2α), angiogenesis (VEGF), and proliferation (Ki-67) were correlated each other and also with patient’s clinicopathological characteristics such as the age at diagnosis, pre-operative Karnofsky Performance Status (KPS), OS and PFS. The Spearman’s rank correlation coefficients are presented in Table 4. Statistically significant results (P < 0.05) are highlighted.

**Table 4:** Mutual correlations among the expression of hypoxia, angiogenesis, microvascular, proliferation markers, and clinical-pathological characteristics (Spearman’s rank-order correlation coefficients)

	KPS	PFS	OS	CD31	CD34	vWF	CD105	VEGF	HIF2	CD105/CD31	CD105/CD34	CD105/vWF	Ki-67
Age	**-**0.076	-0.380^**^	-0.583^**^	**-**0.119	**-**0.105	0.020	0.203	**-**0.009	-0.278^*^	0.306^*^	0.358^**^	0.158	0.004
KPS	-	0.207	0.082	0.054	0.070	0.028	0.025	0.037	**-**0.087	**-**0.094	**-**0.149	**-**0.107	0.097
PFS	-	-	0.781^**^	0.229	0.181	0.082	**-**0.043	**-**0.078	0.179	-0.318^*^	-0.288^*^	**-**0.156	-0.118
OS	-	-	-	0.206	0.210	0.089	0.023	**-**0.124	0.259	**-**0.242	**-**0.232	**-**0.048	-0.073
CD31	-	-	-	-	0.845^**^	0.760^**^	0.482^**^	0.163	0.189	-0.492^**^	-0.405^**^	-0.298^*^	-0.193
CD34	-	-	-	-	-	0.859^**^	0.590^**^	0.270	0.285^*^	-0.300^*^	-0.475^**^	-0.321^*^	-0.143
vWF	-	-	-	-	-	-	0.662^**^	0.189	0.102	**-**0.164	**-**0.258	-0.389^**^	-0.195
CD105	-	-	-	-	-	-	-	0.199	0.072	0.440^**^	0.368^**^	0.374^**^	-0.143
VEGF	-	-	-	-	-	-	-	-	0.330^*^	0.004	**-**0.132	**-**0.049	0.228
HIF2	-	-	-	-	-	-	-	-	-	**-**0.155	-0.317^*^	**-**0.050	0.194
CD105/CD31	-	-	-	-	-	-	-	-	-	-	0.854^**^	0.768^**^	0.112
CD105/CD34	-	-	-	-	-	-	-	-	-	-	-	0.817^**^	0.005
CD105/vWF	-	-	-	-	-	-	-	-	-	-	-	-	0.049

^**^ Correlation is significant at the 0.01 level (2-tailed)

^*^ Correlation is significant at the 0.05 level (2-tailed)

### Relation between microvascular, angiogenesis, hypoxia, and proliferation markers and IDH1 R132H mutation status

The differences in expression of endothelial markers (CD31, CD34, vWF) together with markers of active endothelia (CD105), proportions of newly-formed microvessels (ratios CD105/CD31, CD105/CD34 and CD105/vWF), intratumor hypoxia (HIF2α), angiogenesis (VEGF), and proliferative activity (Ki-67) were examined between GBM samples with IDH1 R132H mutation by comparison with IDH1 wild-type tumors.

The only statistically significant result was observed for VEGF with higher expression in IDH1 wild-type than IDH1 R132H mutated tumors (median 0.02285 vs. 0.017706, respectively; P = 0.01, Mann-Whitney U test).

### Relation between microvascular, angiogenesis, and hypoxia markers and patients’ survival

Patients with GBM expressing endothelial marker CD31 above the median (60.4 mm^-2^) experienced significantly longer estimated median PFS than patients with CD31 bellow this value (64 vs. 43 days, respectively; P = 0.041, long-rank test). Similarly, expression of endothelial marker CD34 above the median (63.4 mm^-2^) was associated with significantly longer estimated median PFS than CD31 bellow this value (61 vs. 48 days, respectively; P = 0.042, long-rank test).

On the contrary, the higher expression of markers assessing the newly-formed microvessels (ratio CD105/CD31) over the median (0.547) was associated with shorter estimated median PFS compared with expression bellow this value (48 vs. 74 days, respectively; P = 0.026, long-rank test).

## DISCUSSION

Recurrent IDH mutations and their role for oncogenesis and tumor progression were described for the first time in GBM [5, 34]. This observation led to new insights into GBM biology with the major role of alterations in cancer cell metabolism for some portion of these tumors [35]. Predominantly secondary GBM that progressed from the low grade tumors were identified to harbor the IDH mutations [36]. The IDH1 and IDH2 mutations were subsequently indentified in the majority of lower grade gliomas, mostly in diffuse astrocytomas (62%), anaplastic astrocytomas (46%), oligodendrogliomas (77%), and anaplastic oligodendrogliomas (73%) [37].

The main oncogenic role of IDH mutations are likely caused by the change in the metabolic activity of this enzyme. Instead of the production of alpha-ketoglutarate, mutated IDH produced oncometabolite 2-hydroxyglutarate (2-HG) that was highly accumulated in cancer cells [11]. Subsequently, 2-HG inhibits the functions of the alpha-ketoglutarate dependent superfamily of dioxygenases that have diverse cellular functions including histone demethylation and demethylation of hypermethylated DNA [38, 39]. IDH mutations and 2-HG production were identified to be the sufficient steps in the process leading to glioma hypermethylator phenotype [40]. This finding was important for understanding of glioma oncogenesis and highlighted the interplay between genomic and epigenomic changes in cancers [41].

The assessment of IDH mutations, predominantly IDH1 codon 132 and IDH2 codon 172 missense mutations, was recently incorporated into the updated 2016 WHO classification of CNS tumors [17]. The so-called “integrated diagnosis” combining the histological tumor typing, tumor grading using the four-tiered WHO grading scheme, and the tissue-based molecular analyses of IDH mutations and other molecular characteristics represents now the standard in the diagnostic process of CNS astrocytic and oligodendroglial tumors, including GBM [17, 18].

From the clinical point of view, the assessment of IDH mutations in gliomas is crucial for the prediction of patient prognosis, which is especially emphasized for GBM. Across a number of studies, the substantial differences in OS between IDH-mutant and IDH wildtype (IDH-WT) GBM were identified such as 3.8 versus 1.1 years [5], 2.6 versus 1.3 years [8], 2.3 versus 1.2 years [9], and 3 years versus 1 year [13]. Similarly, OS differences were observed in patients with IDH-mutant versus IDH-WT anaplastic astrocytomas - 5.4 versus 1.7 years [8], 6.8 versus 1.6 years [9], and 7 versus 2 years [13]. IDH mutations keep their important diagnostic and prognostic role also for patients with low-grade gliomas [7, 42–44]. Recent meta-analysis confirmed the prognostic role of IDH1/2 mutations in gliomas as well [45].

In our study we also proved the presence of IDH1 R132H mutation in the tumor tissue to serve as the positive prognostic factor for patients with GBM in relation to PFS as well as OS. The IDH1 mutation remained positive prognostic factor for OS (HR = 0.433, P = 0.011) but not PFS (HR = 0.560, P = 0.092) in the multivariate analysis as well. However, the differences in median PFS (136 vs. 44 days) and OS (270 vs. 124 days) between patients with and without IDH1 mutated tumors are not as meaningful as in other studies. The reason for this could be a big heterogeneity of treatments applied to our patients in the real clinical practice. The recommended standard treatment protocol with neurosurgery followed by concomitant chemo-radiotherapy with temozolomide [46] was implemented in 15 patients. The radiotherapy only after surgery was provided to other 15 patients. Importantly, 22 patients were treated neither by radiotherapy nor by chemotherapy after the surgery. The treatment after surgery (radiotherapy or radiotherapy plus chemotherapy) compared with the neurosurgery alone served as a strong positive prognostic factor for patients PFS (HR = 0.241, P<0.001) and OS (HR = 0.343, P=0.003) in multivariate analysis as well. Moreover, older patients (age ≥ 60 years) had significantly worse prognosis for OS than younger patients (HR = 3.691, P = 0.002). Both the age at diagnosis and postsurgical treatment with either radiotherapy or radiotherapy plus chemotherapy are known strong prognostic factors for patients with GBM [47–50].

A limited number of patients included into the study can impinge the survival analysis as well, which is caused mainly by low incidence of GBM among other tumor types. Because of a relatively high number of IDH1 R132H mutated tumors (20 from 52 patients, 38.5%) identified in this study, distinction between primary and secondary GBM was done based on clinical information from each patient history, where possible. After that the majority of IDH1 mutated tumors (16 from 18 patients, 88.9%) were classified as secondary GBM that is in accordance with similar studies [8, 36, 51].

GBM is highly vascularized tumor with substantial microvascular proliferation predominantly surrounding necrotic areas [17, 21, 22, 29]. The process of angiogenesis is turned on by the presence of hypoxia that results in upregulation of hypoxia inducible factors (HIF1α and HIF2α), which subsequently leads to the upregulation of vascular growth factors such as VEGF and microvascular proliferation [52–54]. In addition to the essential role of VEGF also other growth factors were identified to play an important role in GBM angiogenesis such as hepatocyte growth factor (HGF), fibroblast growth factor (FGF), platelet-derived growth factor (PDGF), or angiopoietins and interleukin-8 [55–58]. The pathological angiogenesis can be also constitutively stimulated in non-hypoxia dependent manner by the aberrant activation of the main cellular pathways in GBM such as mitogen-activated protein kinase (MAPK) and phosphatidylinositol 3-kinase (PI3K) signalizations [21, 54, 59].

Abundant GBM microvasculature as a response to aberrant angiogenesis was observed in a number of studies, even if the selection of appropriate endothelial marker is an essential factor for the microvessels assessment. The pan-endothelial marker CD34 was expressed in the significant portion of giant cell GBM (73%) as well as non-giant cell GBM (56%) with the strong widespread staining in 55% and 25% of tumors, respectively [60]. The significant relationship between the expression of pan-endothelial marker CD31 and tumor grade was found in the study with 45 astrocytomas where the highest expression was observed in GBM samples (P = 0.001) [61]. Another study examined the degree of angiogenesis in GBM measured by microvessel density (MVD) using both pan-endothelial marker CD31 and marker CD105/endoglin, which preferentially react with active endothelial cells in angiogenic tissue [62]. MVD was correlated with the expression of VEGF and patient prognosis. MVD was increasing with the higher grades of gliomas and the GBM was the most vascularized tumor. MVD assessed by CD105 was more closely correlated with VEGF expression than by CD31 endothelial marker. Moreover, GBM patients showing higher CD105 MVD had a significantly shorter survival than those with lower CD105 MVD tumors (P = 0.0131), which was not the case for CD31 [62]. The study concluded that CD105 may be a better marker than CD31 for evaluation of angiogenesis and prediction of prognosis in astrocytic tumors. The comparison between CD105 and CD31 MVD in the assessment of GBM angiogenesis was examined in another study with 46 tumor samples as well [63]. CD105 MVD was significantly higher than CD31 MVD (median 49 vs. 37 microvessels per field, respectively, P<0.001) and CD105 MVD was more closely correlated with VEGF (R = 0.421, P = 0.003) than CD31 MVD (R = 0.330, P = 0.024). Moreover, patients with lower CD105 MVD had increased survival compared with those with higher CD105 MVD (P = 0.045) that was not the case for CD31 (P = 0.340). More recently, the angiogenesis was examined by the assessment of CD105 and CD31 MVD in addition to VEGF expression in 50 adult GBM patients [63]. CD105 MVD was significantly higher in GBM compared with peritumoral tissue samples (P=0.012), that was not observed for CD 31. However, inside the tumor tissue the positive correlation was found between MVD assessed by CD 105 and CD 31 endothelial markers (R = 0.630, P<0.001). Both CD105 and CD31 MVD significantly correlated with VEGF expression (P<0.001). The large study including 208 GBM samples evaluated tumor angiogenesis by the expression of CD34, PDGF-C, VEGF and CD105 markers and their relation to HIF1α expression [64]. The expression of HIF1α positively correlated with VEGF and PDGF-C expression in tumor samples (P < 0.001). Moreover, endothelial cells expressing PDGF-C and VEGF were also positive for CD105 confirming the proangiogenic effect of these factors in GBM. Very recently, the immunohistochemical expression of CD34 and CD105 was examined in 43 GBM patients of which 20 experienced tumor hemorrhage [65]. Interestingly, tumors that hemorrhaged had higher expression of CD34 (P = 0.0583) and CD105 (P = 0.0467) as well as HIF1α mRNA (P = 0.0073) by comparison with non-hemorrhaged tumors. Therefore increased hypoxia-induced angiogenesis and microvascular formation may play a role in glioblastoma hemorrhage as well. The relationship between the vascularization assessed by histopathology from tumor samples and radiological methods before the surgery was also observed in recent prospective study with 25 newly diagnosed GBM patients [66]. CD105 MVD expression significantly positively correlated with biomarkers related to tumor perfusion assessed by perfusion computed tomography (permeability surface-area product) and dynamic contrast-enhanced magnetic resonance imaging (volume transfer constant) in this study (R = 0.644, P < 0.001 and R = 0.683, P < 0.001, respectively).

In addition to other studies we evaluated multiple pan-endothelial markers (CD31, CD34, and vWF) together with the marker of active endothelia and newly-formed microvessels (CD105/endoglin) in the assessment of GBM microcirculation and tried to find their mutual relationships. Strong correlations were observed among the pan-endothelial markers CD31, CD34, and vWF. The marker of active endothelia CD105 also correlated with CD31, CD34, as well as vWF, however the relationships were weaker (R = 0.482, 0.59, and 0.662 respectively). Therefore the pan-endothelial markers are commutable with one another, whereas the combination of one pan-endothelial marker with marker of active endothelia provides additional information to the analyses of GBM microvascularity. Only a portion of microvessels detected in the tumor tissue are in fact formed by mitotically active endothelial cells (assessed by CD105 expression). On the other hand, the relationship between the tumor microvascularity (assessed by expression of pan-endothelial as well as CD105 markers) and the leading proangiogenic factor VEGF was not observed in our study. The tumor hypoxia was examined by the assessment of expression of hypoxia inducible factor HIF2α, which is more specific than HIF1α for brain tissue and is widely expressed in high grade gliomas including GBM [67, 68]. Even if the VEGF moderately correlated with HIF2α (R = 0.33), there was no relationship between the tumor microcrovascularity and the hypoxia inducible factor HIF2α, with the only exception of a weak positive correlation between HIF2α and CD34 expression (R = 0.285). We observed no relationships among tumor proliferative activity (Ki-67) and tumor microvascularity, angiogenesis, or hypoxia. We also examined the portion of GBM-associated newly-formed microvessels by the assessment of the ratios between the expression of CD105 and pan-endothelial markers (CD105/CD31, CD105/CD34, and CD105/vWF) and their relation to VEGF and HIF2α. Although there were substantial mutual correlations among these ratios, no association to VEGF was found and only moderate negative correlation of CD105/CD34 with HIF2α was observed (R = 0.317). Therefore the process of angiogenesis in our GBM samples could be only very little dependent on the HIF/VEGF axis. It could rather go through the alternative pathways such as the aberrant activation of MAPK, PI3K, or Notch signaling that were also described in GBM [54, 59, 69]. Interestingly, the CD105/CD31 and CD105/CD34 ratios were positively associated with the patient age (R = 0.319 and R = 368, respectively). This indicated that older compared with younger patients had more newly-formed microvessels in their tumors, even though the correlations were relatively weak.

We observed that the expression of CD31 and CD34 above the median levels served as the positive prognostic factor for patient PFS. On the contrary, patients with higher portion of newly-formed microvessels (assessed by the ratio CD105/CD31 above the median level) experienced significantly shorter PFS. Therefore in addition to other studies, we explored that just the portion of newly-formed microvessels in tumor tissue can be a negative prognostic factor for survival of patients with GBM.

The relationship between the presence of IDH mutations and tumor angiogenesis has been also studied, though with inconclusive results. As discussed above, HIF1α and HIF2α have the central role in cellular response to hypoxia and initiating of VEGF-mediated angiogenesis. The activity of hypoxia inducible factors is negatively regulated by the prolyl hydroxylase (PHD) that promote HIF degradation by the hydroxylation of two proline residues in its oxygen-dependent degradation domain, which in turns leads to association of HIF with the pVHL E3 ligase complex and its degradation via the ubiquitin-proteasome pathway [70]. PHD belongs to the alpha-ketoglutarate dependent dioxigenases. Therefore it was hypothesized that the aberrantly produced 2-HG by mutated IDH may compete with alpha-ketoglutarate and inhibit PHD, which in turn can lead to the pathological stabilization of HIF, induction of VEGF expression and thus initiation of tumor angiogenesis [23, 24]. However, subsequent studies did not prove this hypothesis when no significant correlation was observed between IDH mutations and HIF1α expression in glioma samples [25, 26]. Above that, more recent studies in human astrocytes, colorectal and erythroleukemia cell lines found the negative association between IDH mutations and HIF1α expression caused by the 2-HG-dependent stimulation rather than inhibition of PHD [27, 28].

We also examined if there was a difference in expression of hypoxia inducible factor (HIF2α) as well as its downstream pro-angiogenetic factor VEGF in accordance to IDH1 R123H mutation status in our GBM samples. In addition to studies discussed above, we examined whether there was a difference in microvessels formation between the IDH1 mutated and IDH1 wild-type tumors as well. We found that HIF2α expression and tumor microvessels formation were independent of the IDH1 mutation status. Moreover, IDH1 mutated tumors experienced in fact decreased expression of VEGF by comparison to IDH1 wild-type GBM. Finally, the tumor proliferative activity assessed by the expression of Ki-67 antigen did not differ with the IDH1 mutation status.

The main limitation of the presented study is a relatively low number of included patients as well as the substantial heterogeneity in applied postsurgical treatment that can impinge the survival analysis. Also the mechanism study is needed to elucidate the role of IDH1 mutation in GBM angiogenesis and neovascularization, which is considered for designing the follow-up projects.

In summary, our results did not prove the hypothesis that angiogenesis in IDH-mutated GBM is caused mainly by IDH mutation-dependent expression of HIF and downstream overexpression of VEGF. Rather than VEGF-mediated angiogenesis, the alternative mechanisms of microvessels formation such as the aberrant activation of MAPK, PI3K, or Notch signaling can play a significant role in our GBM tumors. We also showed that the combination of pan-endothelial marker together with the marker of active endothelia CD105/endoglin can improve the histological assessment of GBM microvasculature and better predict of patient’s prognosis.

## MATERIALS AND METHODS

### Patients

52 patients with a diagnosis of GBM (n = 52; 28 males and 24 females; median age 64.3 years) who were treated (tumor resection, radiotherapy, and chemotherapy with temozolomide) in the Faculty Hospital in Pilsen between the years 2009 and 2014 were included into the retrospective study (Table 5). The study protocol was approved by the ethics committee.

**Table 5:** Characteristics of glioblastoma patients included in the study

Patient characteristics	
**Sex**	
Male	28
Female	24
**Age, years**	
Median	67
Range	35 - 87
**KPS**	
Median	80
Range	30 - 100
**Postoperative treatment**	
RT + CT	15
RT alone	15
None	22

*KPS* - Karnofsky performance score; *RT* - radiotherapy; *CT* - chemotherapy

### DNA isolation

DNA was extracted from 10 μm sections of formalin-fixed, paraffin-embedded (FFPE) tissue samples following macro dissection of tumor tissue and normal brain tissue using the QIAamp DNA FFPE Tissue kit (Qiagen, Hilden, Germany). The 10 μm sections corresponded to haematoxylin and eosin representative with tumor tissue verified by a pathologist specialized in brain tumors.

### Mutation detection

For detection of mutant allele IDH1 c.395G>A (p.R132H, COSMIC ID 28746), TaqMan Mutation Detection Assays (assay name: IDH1 28746mu and IDH1 rf) with the TaqMan Mutation Detection IPC Reagent Kit (Life Technologies, Carlsbad, California, USA) were used. Mutant allele detection was performed according to recommended procedure and reaction conditions in the manual. For the amplification, the Stratagene Mx3000P realtime PCR system instrument (Agilent Technologies, Inc., Santa Clara, CA, USA) was used. Detection of mutant alleles was performed in duplicate in a reaction volume of 20 μL. Detection of reference gene was also performed in duplicate. The analysis of positive samples was repeated. Before analysis of collection of tumor samples, the samples of normal brain tissue were analyzed for detection of cut-off amplification curve. No amplification of mutant allele was present in normal brain tissue. On the basis of these results and the shape of amplification curve of positive tumor samples, the 25 ΔCt cut-off value was determined.

### Histological processing and quantification

Tissue FFPE samples for light microscopy were cut into 5 μm sections and stained with hematoxylin and eosin. Adjacent sections were processed immunohistochemically using Ventana Benchmark XT automated stainer (Ventana Medical System, Inc., Tucson, AZ, USA) with diaminobenzidine visualization and counterstaining with Mayer’s haematoxylin. The details on monoclonal primary antibodies employed together with appropriate positive controls are listed in Table 6. Briefly, three markers were used to detect the endothelial cells lining the tumorous blood vessels (CD34, CD31, and vWF) and another marker (CD105) was used for newly-formed proliferation-associated endothelial cells. Stimulation of angiogenesis was assessed using the anti-VEGF-A (vascular endothelial growth factor A - VEGF) antibody. Tissue hypoxia and possible regulation of the VEGF expression was assessed using the anti HIF2α (type 2 hypoxia inducible factor) antibody. The proliferative activity of the tumor was assessed by the expression of Ki-67 antigen.

**Table 6:** Immunohistochemical reagents for the histological analysis

Primary antibody, clone and dilution	Manufacturer	Used for detection of
Monoclonal Mouse Anti-Human CD34 Class II, Clone QBEnd 10 (Dako), 1:30	DakoCytomation, Glostrup, Denmark	endothelial cells
Monoclonal Mouse Anti-Human CD31 , Endothelial cell, Clone JC70A, 1:40	DakoCytomation, Glostrup, Denmark	endothelial cells
Polyclonal Rabbit Anti-Human Von Willebrand Factor (vWF), 1:200	DakoCytomation, Glostrup, Denmark	endothelial cells
Monoclonal Mouse Anti-Human CD105, Endoglin, Clone SN6h, 1:5	DakoCytomation, Glostrup, Denmark	proliferation-associated endothelial cells
Polyclonal Rabbit Anti-Human VEGF-A (vascular endothelial growth factor), 1:100	Zytomed Systems, Berlin, Germany	up-regulation of angiogenesis
Monoclonal Mouse Anti-Human HIF2α (hypoxia inducible factor), Clone ep190b, 1:30	Abcam, Cambridge, UK	tissue hypoxia and regulation of VEFG expression
Monoclonal Mouse Anti-Human Ki67, Clone MIB-1, 1:400	Dako, Glostrup, Denmark	proliferation-associated antigen

**Figure 3:**
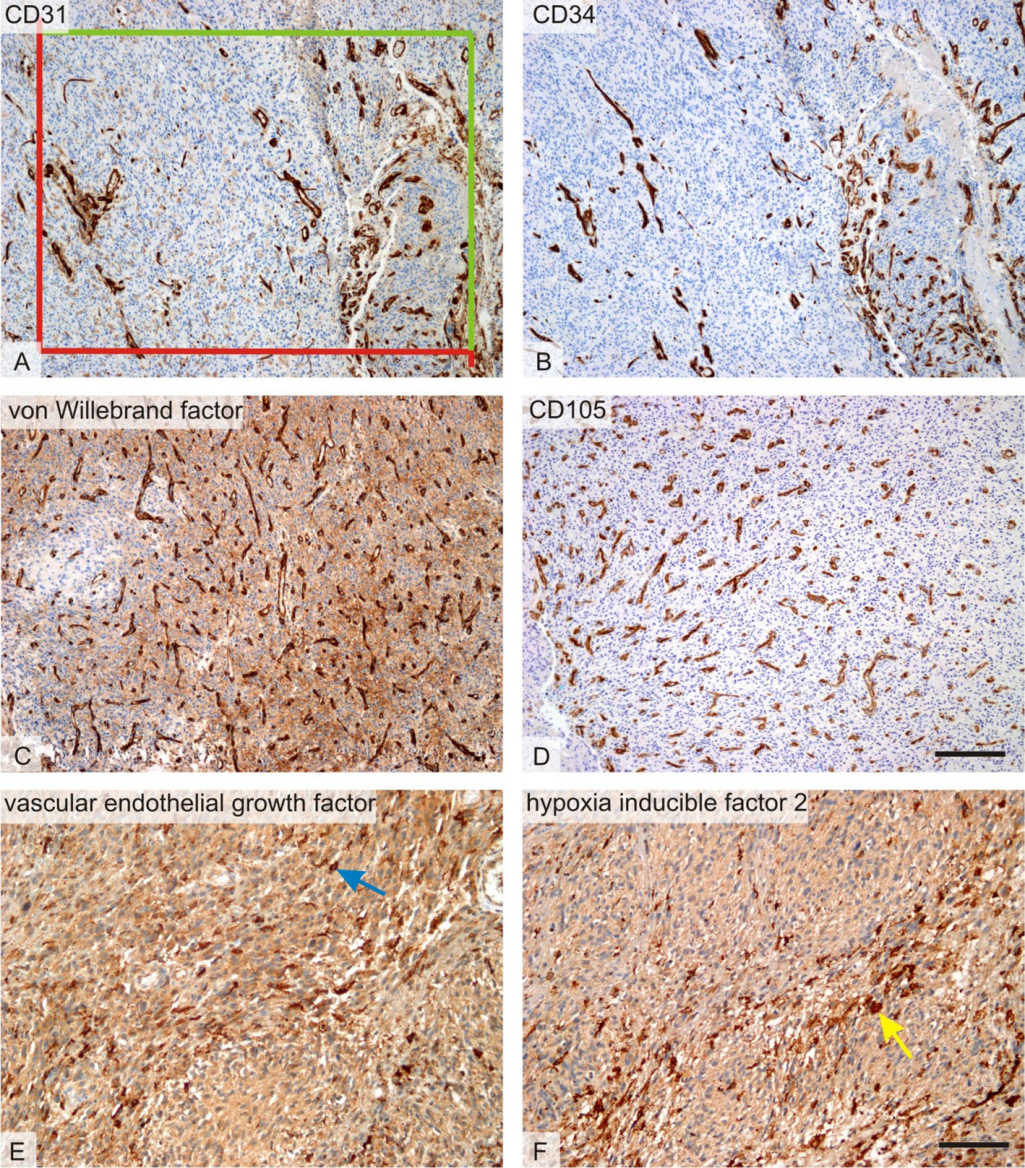
Histological markers of angiogenesis Micrographs representing the six methods used for quantitative assessment of microvascular bed and its regulation within the glioblastoma tumors. **(A)** Microvessels with CD31-positive endothelium. In all endothelial markers, the number of microvessel profiles per section area was counted using a projection of unbiased counting frame consisting of two admittance (green) and two forbidden (red) borders. **(B)** Microvessels with CD34-positive endothelium on a micrograph representing an area on a serial section corresponding to figure A. **(C)** Microvessels with endothelium positive to von Willebrand factor (vWF). In most glioblastoma samples, von Willebrand factor was less readable due to a slightly positive background when compared to CD31 of CD34 immunohistochemistry. **(D)** Microvessels with proliferation-associated endothelium positive to von CD105. The micrograph was taken from an area corresponding to that on the B. **(E)** Vascular endothelial growth factor (VEGF)-positive cells (blue arrow) and (**F)** hypoxia inducible factor 2 (HIF-2)-positive cells (yellow arrow) were frequently found close to the necrosis (necrotic areas were excluded from the quantification). Positive cells were quantified using their area fraction within the tumor. Immunohistochemical detection of the CD31 (A), CD34 (B), von Willebrand factor (C), CD105 (D), VEGF(E), and HIF-2 (F), visualization of the immunoreaction with diaminobenzidine (dark brown), counterstaining with hematoxylin. Scale bar 200 μm (A-D) and 100 μm (E-F).

All quantitative estimates were done using stereological methods and the Ellipse software (ViDiTo, Kosice, Slovakia). Stereological methods used in the study are based on counting intersections of the structures of interest with stereological grids [71] or counting frames [72] randomly superposed on the micrographs. During the histological evaluation, the observers had no knowledge of the biological status of the samples or the patient histories. Magnification and sampling of microscopic image fields per section used for estimating all the quantitative parameters are summarized in Table 7. The borders of the glioblastoma tumor were outlined in sections stained with haematoxylin-eosin and the borders were respected in all the consecutive sections stained by immunohistochemistry. Non-tumorous brain tissue and necrotic areas were excluded from the quantification. The microvascular bed was quantified as the number of CD31, CD34, vWF, of CD105-positive microvessel profiles per section area *QA* of the vascular wall [73, 74]. Unbiased counting frames with known area were projected on micrographs and profiles of microvessels inside the frames or touching the admittance borders but not touching the forbidden lines were counted (Figure 3).

**Table 7:** Sampling of histological sections and microscopic image fields for quantification

Quantitative parameter (component, reference space)	Microscope objective used	Image fields sampled per patient	Number of counting events per patient
Q_A_(CD34): number of CD34-positive microvessel profiles per section area	10×	4	307 vascular profiles counted on average
Q_A_(CD31): number of CD31-positive microvessel profiles per section area	10×	4	331 vascular profiles counted on average
Q_A_(vWF): number of vWF-positive microvessel profiles per section area	10×	4	289 vascular profiles counted on average
Q_A_(CD105): number of CD105-positive microvessel profiles per section area	10×	4	162 vascular profiles counted on average
A_A_(VEGF, tumor): area fraction of VEGF-immunopositive cell profiles	20×	8	>150 intersections with VEGF-positive areas, 204 on average
A_A_(HIF2α, tumor): area fraction of HIF2α -immunopositive cell profiles	20×	8	>150 intersections HIF2α -positive areas, 237 on average

*AA(component, space)* – area fractions of the respective components within their reference spaces; *QA* – number of microvessel profiles per section area. The microscope objective and magnification used for the quantitative assessment of each of the parameters was at the lowest setting that permitted an exact and unambiguous identification of the counting events with respect to histological staining methods. The number of counting events per sample is provided, and the resulting data are presented as arithmetic means of all image fields representing each patient.

The presence of cells expressing VEGF-A and HIF2α was quantified as area fractions A_A_ of their positive immunostaining within the histological section of the glioblastoma tissue. When estimating the area fraction of the detected structures, points of a stereological point grid were superimposed on the micrographs. The area fraction was calculated as a ratio between the number of points hitting the immunopositive cell profiles and all the points hitting the reference area of the glioblastoma tissue. The proliferative activity of the tumor tissue was determined as percentage of MIB-1 stained nuclei per total number of nuclei in four representative cross-sections in each tumor sample.

### Statistical analysis

Statistical analyses were carried out using the SPSS 22 software package (IBM, Armonk, NY). Spearman’s rank-order correlation method was used for mutual correlations of expression of hypoxia, angiogenesis, and microvascular markers with clinical-pathological characteristics. The Mann-Whitney U test was used for the determination of differences between markers in IDH1 mutated compared with IDH1 wild type tumors. Overall survival (OS) was defined as the time between the diagnosis and death or last follow-up. Progression-free survival (PFS) was defined as the time between the diagnosis and recurrence or last follow-up. Survival time was estimated by Kaplan-Meier analyses and compared among patient subsets using log-rank test. Multivariate analysis was performed with the Cox regression model to test independent significance while adjusting for covariates, data are presented as hazard ratios (HR) and 95% confidence intervals (95%CI). Reported P-values were two-sided. P-values of less than 0.05 were considered to indicate statistical significance.

## Abbreviations

2-HG: 2-hydroxyglutarate
CD: cluster of differentiation
EANO: European Association for Neuro-Oncology
FFPE: formalin-fixed, paraffin-embedded
FGF: fibroblast growth factor
GBM: glioblastoma multiforme
HGF: hepatocyte growth factor
HIF1α: hypoxia inducible factor 1 alpha
HIF2α: hypoxia inducible factor 2 alpha
IDH: isocitrate dehydrogenase
IDH-WT: IDH wildtype
IHC: immunohistochemistry
KPS: Karnofsky performance status
MAPK: mitogen-activated protein kinase
mRNA: messenger RNA
MVD: microvessel density
NADP: nicotinamide adenine dinucleotide phosphate
OS: overall survival
PDGF: platelet-derived growth factor
PFS: progression-free survival
PHD: prolyl hydroxylase
PI3K: phosphatidylinositol 3-kinase
pVHL: von Hippel-Lindau protein
RT-PCR: real-time polymerase chain reaction
VEGF: vascular endothelial growth factor
vWF: von Willebrand factor
WHO: World Health Organization
